# Revelation of Inherently High Mobility Enables Mg_3_Sb_2_ as a Sustainable Alternative to n‐Bi_2_Te_3_ Thermoelectrics

**DOI:** 10.1002/advs.201802286

**Published:** 2019-07-13

**Authors:** Xuemin Shi, Cheng Sun, Zhonglin Bu, Xinyue Zhang, Yixuan Wu, Siqi Lin, Wen Li, Alireza Faghaninia, Anubhav Jain, Yanzhong Pei

**Affiliations:** ^1^ Interdisciplinary Materials Research Center School of Materials Science and Engineering Tongji University 4800 Caoan Road Shanghai 201804 China; ^2^ Lawrence Berkeley National Laboratory 1 Cyclotron Road Berkeley CA 94720 USA

**Keywords:** grain size, high mobility, Mg_3_Sb_2_ alloys, thermoelectrics

## Abstract

Over the past years, thermoelectric Mg_3_Sb_2_ alloys particularly in n‐type conduction, have attracted increasing attentions for thermoelectric applications, due to the multivalley conduction band, abundance of constituents, and less toxicity. However, the high vapor pressure, causticity of Mg, and the high melting point of Mg_3_Sb_2_ tend to cause the inclusion in the materials of boundary phases and defects that affect the transport properties. In this work, a utilization of tantalum‐sealing for melting enables n‐type Mg_3_Sb_2_ alloys to show a substantially higher mobility than ever reported, which can be attributed to the purification of phases and to the coarse grains. Importantly, the inherently high mobility successfully enables the thermoelectric figure of merit in optimal compositions to be highly competitive to that of commercially available n‐type Bi_2_Te_3_ alloys and to be higher than that of other known n‐type thermoelectrics at 300–500 K. This work reveals Mg_3_Sb_2_ alloys as a top candidate for near‐room‐temperature thermoelectric applications.

## 1 Introduction

Thermoelectric (TE) technology, which directly converts heat into electricity and vise‐versa based on either Seebeck effect or Peltier effect, has been long attractive for both power generation and refrigeration, though commercial use of this technology is so far limited for niche applications. The materials' thermoelectric figure of merit, *zT* = *S* ^2^ *T*/*ρ* (κ_E_ + κ_L_) is an essential parameter measuring the conversion efficiency, where *S* is the Seebeck coefficient, ρ is the electrical conductivity, *T* is the absolute temperature, and κ_E_ and κ_L_ are the electronic and lattice components of the thermal conductivity, respectively.^1^

With decades of development, there are many effective strategies to enhance *zT*. Typical thermal strategies focus on a minimization of κ_L_ through defect and microstructure engineering for phonon scattering^2^ as well as structural, bonding, and anharmonicity designs for intrinsically low lattice thermal conductivity due to various mechanisms.^3^ Alternatively, electronic strategies typified by band engineering^4^ for an effective decoupling among the strongly correlated *S*, ρ, and κ_E_ are proven to be successful as well, providing the carrier concentration is optimized.^5^ The utilization of these strategies has led to significant advancements mostly in conventional thermoelectrics such as PbTe,[qv: 4a,6] PbSe,[qv: 2c,7] Bi_2_Te_3_,^8^ SiGe,^9^ SnTe,^10^ GeTe,^11^ and Te,^12^ where the toxicity or the scarcity of the constituents are always concerned for sustainable applications.

For low and intermediate temperature applications (< ≈500 K), Bi_2_Te_3_ and its alloys in both n‐ and p‐type conductions are so far the only one family of materials being commercialized,[qv: 8a,13] and alternative materials showing a competing thermoelectric performance in this temperature range have been barely developed. Therefore, it has been a long anticipation in this field for the discovery of such alternatives, particularly with less toxic but more abundant constituents, mainly because of the scarcity of Te.

Richness in composition, chemical, and physical properties of semiconducting Zintl compounds,^14^ largely motivates the development of Zintls for thermoelectric applications,^15^ among which Mg‐based materials (including Mg_2_Si‐,^16^ Mg_3_Sb_2_‐,^17^ and MgAgSb‐based^18^) are of great potential because of the sustainability of Mg. However, the high vapor pressure, causticity of Mg, and the high melting point of Mg_3_Sb_2_ usually lead to difficulties on synthesis, and the resultant defects and impurity phases might be detrimental for thermoelectric performance.^19^

The existence of multiple conduction bands with high valley degeneracies^20^ as well as the allowance of chemical substitutions[qv: 14a] in Mg_3_Sb_2_, leading this material particularly in n‐type to be very interesting for thermoelectric applications, due to the high power factor (≈20 µW cm^−1^ K^−2^[qv: 19a]) and the low lattice thermal conductivity (≈0.6 W m^−1^ K^−1^ 20) in alloys. n‐type Mg_3_Sb_2_ usually involves charge scattering by other sources including ionized impurities[qv: 19a,b,21] and boundary potentials.^22^ Existing challenges on realizing high thermoelectric performance in n‐Mg_3_Sb_2_ are typified by a successful doping for a high enough carrier concentration^23^ and an exclusion of these scattering sources. The latter can be achieved by controlling the composition [qv: 21a,22b,24] and sintering conditions[qv: 19a] as well as by coarsening the grains,^22^ all of which have resulted in ineffective improvements in mobility.

Very recent works have shown that a big grain size is particularly helpful for a high carrier mobility thus a high thermoelectric performance in this material.[qv: 22b,c] Materials with small grain sizes tend to show a thermally activated mobility, which is a signature of detrimental scattering that limits the thermoelectric performance near room temperature. Such a sensitive influence of grain sizes on carrier mobility might be related to the grain surface oxidation in this material that contains a high concentration of caustic Mg, although a direct observation of such a surface oxidation might be challenging. This seems to be consistent with the finding that materials sintered at a higher temperature for longer tend to show a better conductivity.[qv: 19a]

Available literatures have shown a great improvement in n‐type thermoelectric Mg_3_Sb_2_, unfortunately promising thermoelectric performance is so far achievable only at *T* > ≈500 K,[qv: 17a,20,21,23] which can be understood because the scattering of carriers which due to grain boundaries and defects is weakened at high temperatures. Therefore, the current work is motivated to focus on the realization of high thermoelectric performance at *T* < ≈500 K of n‐Mg_3_Sb_2_, through a development of synthesis technique for coarse grains with minimal oxidation.

Briefly, a melting process is applied for coarse grains with good purity, crystallinity, and homogeneity, where a tantalum‐sealing technique is used. The following densification was done by sintering/fusing big pieces (≈3 mm in size) of ingots, for large grains with minimal oxidation in property‐measureable samples. Such a sintering procedure is essentially very similar to hot‐deforming. Importantly, a control experiment of grain size and oxidization reveals that coarse grains with minimal oxidation are indeed helpful for realizing an inherently high carrier mobility in this material. Furthermore, it is shown that Mg_3_Bi_2_‐alloying not only reduces the lattice thermal conductivity and promotes the doping effectiveness for a high carrier concentration but also optimizes the electronic transport properties. Eventually, this work realizes in Mg_3_Sb_2_ materials with coarse grains and minimal oxidation an electron mobility significantly higher than that ever reported. This largely guarantees a very promising thermoelectric performance in optimal compositions at *T* < ≈500 K, which is highly competitive to that of n‐Bi_2_Te_3_ materials. The current work might pave an avenue for Mg_3_Sb_2_ materials, as a sustainable solution to n‐type Bi_2_Te_3_ thermoelectrics.

## 2 Results and Discussion

**Figure 1:**
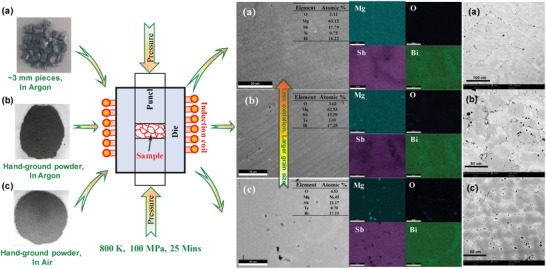
SEM images and the corresponding composition mappings by EDS taken from the polished surface (with and without HNO_3_‐etching) of Mg_3.05_SbBi_0.97_Te_0.03_ pellets hot pressed using a) big pieces, and hand‐ground powders b) in argon‐filled glovebox and c) in air, showing an increase in oxidation but a decrease in average grain size from (a) to (c).

The powder X‐ray diffraction (XRD) patterns and lattice parameters for Mg_3.05_Sb_2−_ *_x_*_−_ *_y_*Bi*_y_*_−_ *_x_*Te*_x_* (*x* ≤ 0.04, *y* ≤ 1.5) samples are shown in Figure S1 and Table S1 in the Supporting Information. The observed diffraction peaks can be nicely indexed to those of Mg_3_Sb_2_ with a CaAl_2_Si_2_ structural type having a space group of *P3m*1,[qv: 14c,17c,25] indicating the high phase purity in this work. In order to obtain materials with coarse grains and minimal oxidation, a tantalum‐sealing technique is used for melting/annealing and a sintering technique (by an induction‐heating hot press system, **Figure** **1**) is developed using big pieces of ingots (≈3 mm in size, Figure 1a). In addition, hot press using powders (dozens of micrometers in size) prepared in an argon‐protected glovebox (Figure 1b) and in air (Figure 1c) is also carried out for different levels of oxidation for the high‐performance composition (Mg_3.05_Sb_1−_ *_y_*Bi*_y_*_−0.03_Te_0.03_).

**Figure 2:**
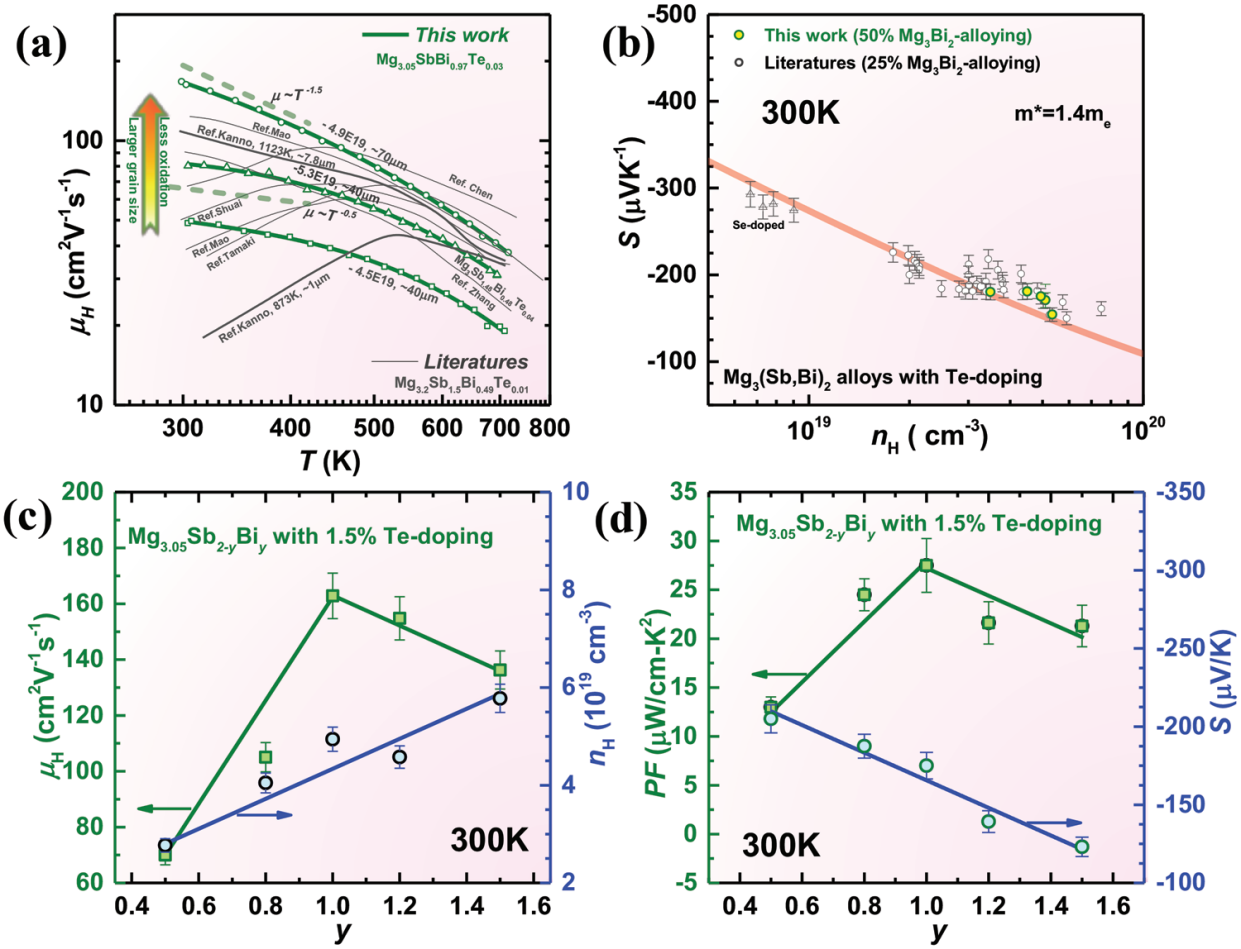
a) Temperature dependent Hall mobility, b) carrier concentration dependent Seebeck coefficient at 300 K, c) room temperature Bi‐alloying dependent Hall mobility and carrier concentration, as well as d) Seebeck coefficient and power factor for Mg_3.05_Sb_2−_ *_x_*_−_ *_y_*Bi*_y_*_−_ *_x_*Te*_x_* (*x* ≤ 0.04, *y* ≤ 1.5) with a comparison to literature results.[qv: 17a,19,20,22a,b] The unit of carrier concentration identifying samples is cm^−3^.

Detailed scanning electron microscope (SEM) observations (Figure 1) on the polished and HNO_3_‐etched surface of the pellets are carried out, to determine the average grain size. The optimal etching condition that well balances the boundary and in‐grain corrosions is determined to be a surface coating with 0.6 wt% aqueous HNO_3_ solution for 20 s at room temperature. More details on the statistical estimation of circle equivalent diameter of grains are given in Figure S2 and Table S2 in the Supporting Information. The resulting SEM images enable a reasonable determination of statistic average circle equivalent grain diameter of ≈60 µm for the hot‐deformed sample starting with big pieces (Figure 1a) and ≈30 µm for the hot‐pressed samples starting with powders (Figure 1b,c). As one can see, the room temperature mobility increases from ≈20 to ≈100 cm^2^ V^−1^ s^−1^ once the average grain size increases from 1 to 8 µm respectively synthesized by spark plasma sintering (SPS) at 873 and 1123 K.[qv: 22b,c] With a further increase in the average grain size to ≈60 µm (Figure 1a) in this work, the room temperature mobility continuously increases to ≈160 cm^2^ V^−1^ s^−1^ (**Figure** **2**a). This suggests that the grain size indeed takes the responsibility for the high mobility in this work.

According to the energy dispersive spectrometer (EDS) analyses, it is shown that the oxygen content increases from 2.1% to 3.6% and then to 4.5% (Figure S1a–c, Supporting Information), as the exposure to air increases (Figure 1a–c). Importantly, once the oxygen content increases from 3.6% to 4.5% in samples with a similar average grain size of ≈30 µm, the mobility decreases from 85 to ≈50 cm^2^ V^−1^ s^−1^, which indicates the detrimental effects of grain surface oxidation in terms of mobility. According to the recent literatures,^22^ the potential barriers at boundaries play a very important role on charge scattering in this material particularly at *T* < ≈500 K, indicating the possibility that an oxidation‐free grain surface would be helpful for a high mobility. This seems to be consistent with the current work that materials with surface oxidation of grains indeed show lower mobilities, as compared to that of literature results on a material with an average grain size of 8 µm,[qv: 22b,c] although the average grains size of ≈30 µm is much larger.

It is interesting to note that a few literature works have shown that the existence of cation doping[qv: 17a,19b,21a] and a high temperature (1123 K[qv: 19a,22b]) sintering process is effective for increasing the mobility by weakening the charge scattering due to ionized impurities (such as Mg vacancies). Alternatively, it is shown that a high sintering temperature of 1123 K enables an effective increase in grain size and the resultant coarse grains are claimed to take the responsibility for the increase in mobility observed.[qv: 22b,c] This work involves no intentional doping at the cation site and the hot‐pressing temperature of 800 K used in this work is much lower, therefore, the current results seem to be consistent with the general argument of coarse grains for high mobilities.

It should be kept in mind that a high temperature sintering process not only tends to coarsen the grains in general but also favors the purification of grain boundaries due to the grain growth, and both coarse grains and clean boundaries are alternatively realized in this work by sintering/deforming large pieces of ingot material (Figure 1). More quantitatively on temperature dependent mobility (Figure 2a), with a decrease in grain size and an increase in grain surface oxidation, the scattering parameter *r* according to the relationship μ_H_ ∼ *T^  r^* at *T* < ≈500 K deviates from −1.5 for a pure acoustic scattering mechanism to −0.5 for a mixed scattering mechanism. Therefore, this work indicates that coarse grains with minimal surface oxidation would lead to a high mobility, presumably due to the reduced boundary concentration and boundary potential barriers.

It is interesting to note that this work reveals a substantially high electron mobility (Figure 2a) than that ever reported,[qv: 17a,19,20,22b] with the synthesis technique developed. In addition, materials synthesized in this work all show a dominant charge scattering by acoustic phonons, which is consistent with that of largely grained materials in the literatures.[qv: 17a,22b,c] Therefore, this work enables a synthesis approach revealing the inherently high electron mobility of n‐type Mg_3_Sb_2_‐based thermoelectrics.

**Figure 3:**
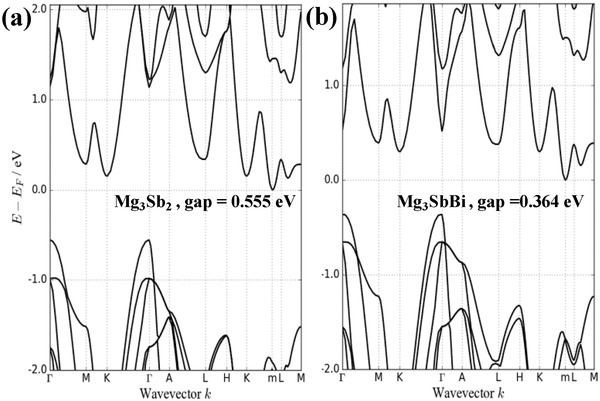
Calculated band structures for a) Mg_3_Sb_2_ and b) Mg_3_SbBi. Note that the CBM is located inside Brillouin zone (BZ) close to *L* point. This point (*l*) is not one of the high symmetry points of the BZ. The fermi surface is available in Figure S3 in the Supporting Information.

The high thermoelectric performance in n‐type Mg_3_Sb_2_‐based materials largely stems from its highly degenerated transporting conduction band,^20^, ^26^ as confirmed by our band structure calculations (**Figure** **3**; details on band structure calculations are given in the Supporting Information). The high band degeneracy comes from the existence of highly equivalent symmetric operations of band minima along a low‐symmetry direction of *L* to *M* in the Brillouin zone of the highly symmetric crystal structure of Mg_3_Sb_2_ (space group number = 164), this leads an overall valley degeneracy to be as high as 6. The Fermi surface at 0.1 eV above the conduction band minima shows 6 in total roughly spherical electron pockets, indicating the charge transport can be approximated as a single parabolic band behavior with 6 degenerated isotropic valleys. Such a high valley degeneracy leads to a relatively high density of states mass (m*) of 1.4 *m*_e_ (Figure 2b), as compared to that of conventional n‐type thermoelectrics including Bi_2_Te_3_ (≈0.60 *m*_e_ 27) and PbTe (≈0.26 *m*_e_[qv: 5c]). This leads to a higher carrier concentration required for maximizing the thermoelectric performance.[qv: 5c,28]

A realization of a sufficiently high electron concentration in Mg_3_Sb_2_‐based thermoelectrics is challenging,^29^ and Te‐doping[qv: 20,29b] for anions while Mn‐doping[qv: 17a] for cations are known to be effective. According to the literature studies,[qv: 17a,22a,29a,30] the defect formation energy of Te/Sb substitutions is lower than that of Te/Sb ones and Mg interstitials. This leads to a high doping efficiency of Te for a sufficiently high electron concentration, while Te‐doping does not show a clear contribution to the mobility. It is suggested that an excess of Mg (usually ≈7%) is helpful for a high doping efficiency,[qv: 19c] as done in this work but we require a much less quantity (≈1.7%) resulting in less impurities. In addition, literature work[qv: 19a,22a,29a] enabling a high electron concentration for a high thermoelectric performance usually involves Mg_3_Bi_2_‐alloying, suggesting an increase in Te‐dopability due to Mg_3_Bi_2_‐alloying. We confirm that an increase of Mg_3_Bi_2_‐alloying at a given Te‐doping of 1.5% leads to a continuous increase in electron concentration (Figure 2c) hence a continuous decrease in Seebeck coefficient (Figure 2d).

The variation of Mg_3_Bi_2_‐alloying in this work further enables an estimation of composition (≈50%) maximizing the power factor (Figure 2d) at room temperature, which is consequently selected as the focus aiming at a high performance at *T* < 500 K in this work. Furthermore, such an alloy composition of ≈50% is found to be optimal for thermoelectric performance as well (Figure S4, Supporting Information), which is different from the composition (≈25%) that is usually focused on in the literatures.[qv: 19c,26] This further motivates an in‐depth study on Mg_3_SbBi alloys with Te‐doping, where the detailed temperature dependent properties are given in Figure S5 in the Supporting Information.

**Figure 4:**
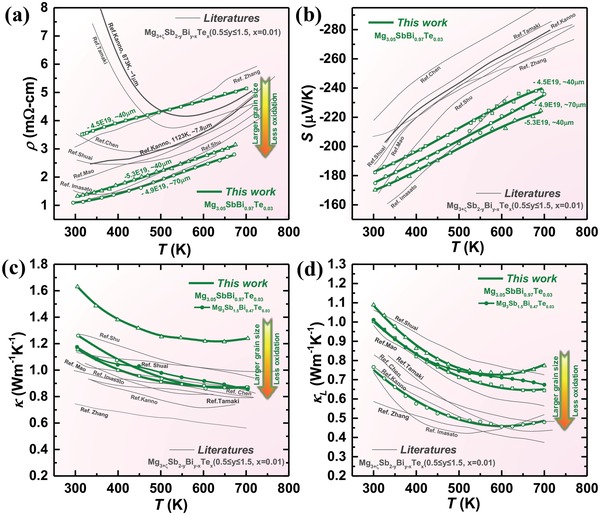
a) Temperature dependent resistivity, b) Seebeck coefficient, and c) total and d) lattice thermal conductivity for Mg_3.05_Sb_2−_ *_x_*_−_ *_y_*Bi*_y_*_−_ *_x_*Te*_x_* (*x* ≤ 0.04, *y* ≤ 1.5) with a comparison to literature results.[qv: 17a,19,20,22a,b,24b] The unit of carrier concentration identifying samples is cm^−3^.

The high mobility and carrier concentration guarantee a low resistivity (**Figure** **4**a). As compared to the literature results,[qv: 19a,20,22a,b] the temperature dependence of resistivity in this work is consistent with that of conventional high‐performance thermoelectrics while in a few literatures,[qv: 17a,19b,c,24b] a carrier scattering mechanism deviating from a pure acoustic scattering leads to abnormalities[qv: 21b]in temperature‐resistivity dependency. This indicates that coarse grains with minimal grain surface oxidation might help a revelation of inherently superior electronic performance in n‐type Mg_3_Sb_2_ thermoelectric alloys. The highly degenerated transporting band (the one along the *L*–*M* direction, Figures 3 and 2b) ensures a high Seebeck coefficient (Figure 4b) in the entire temperature range. Both Seebeck coefficient and resistivity increase with increasing temperature, indicating a degenerate semiconducting behavior. With the measured total thermal conductivity (Figure 4c), the lattice thermal conductivity (Figure 4d) can be estimated by subtracting the electronic component from the total thermal conductivity (κ) according to the Wiedemann–Franz law, where the Lorenz factor is determined by a single parabolic band approximation with acoustic scattering.^31^

**Figure 5:**
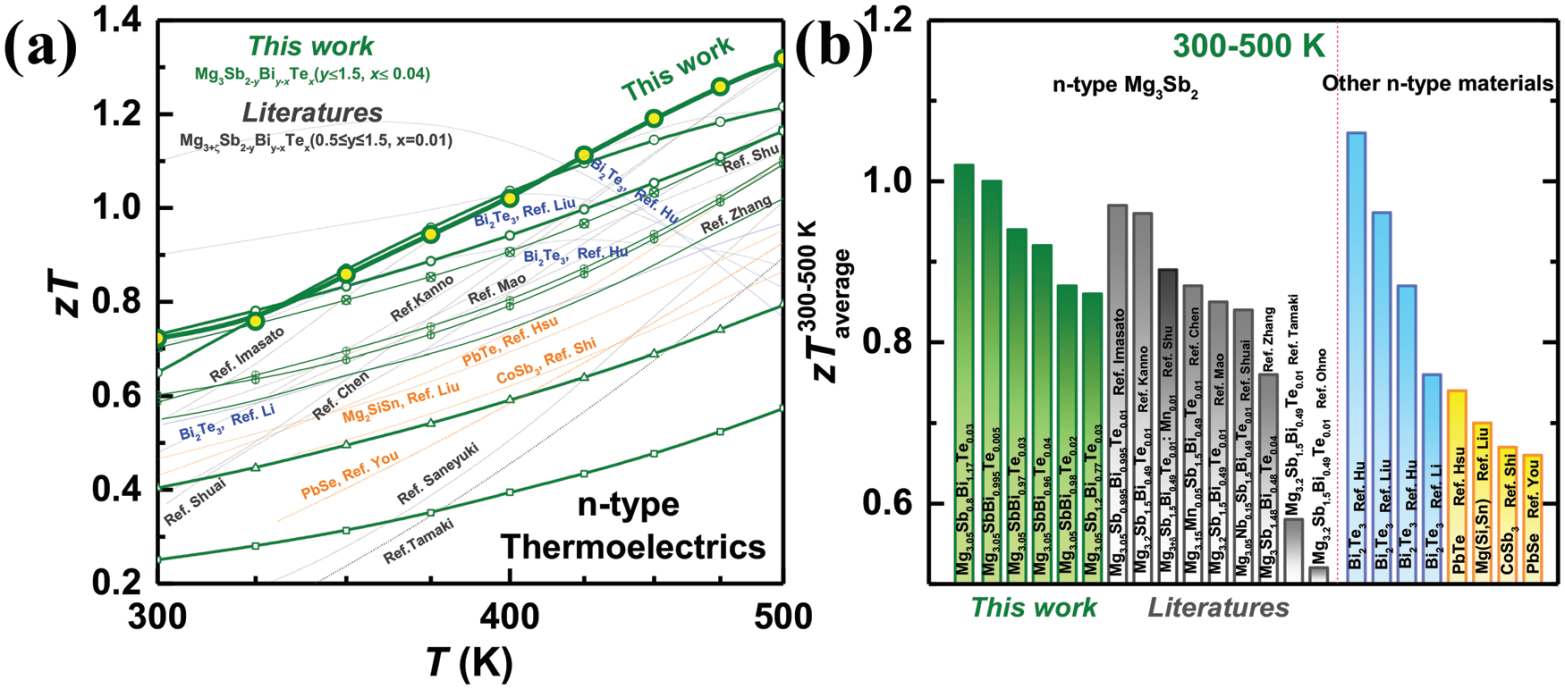
a) Temperature dependent thermoelectric figure of merit, *zT* and b) its average in temperature range of 300–500 K for Mg_3.05_Sb_2−_ *_x_*_−_ *_y_*Bi*_y_*_−_ *_x_*Te*_x_* (*x* ≤ 0.04, *y* ≤ 1.5), with a comparison to literature results for n‐type thermoelectrics.[qv: 7b,8b,16c,17a,19,20,22a,b,29a,33]

As can be seen, Mg_3_Bi_2_‐alloying not only improves the Te‐dopability, but also reduces the lattice thermal conductivity (κ_L_) due to the additional phonon scattering by mass and strain contrasts of anion substitutions. It should be noted that literature works usually focus on a composition of 25% Mg_3_Bi_2_‐alloying,[qv: 19c,26] which should have a higher κ_L_ than that in this work. Moreover, materials obtained in this work have large grains, where the phonon scattering due to grain boundary would be weak. In addition, once there, the high‐κ_L_ oxide (≈60 W m^−1^ K^−1^ at 300 K^32^) could lead to an increase in κ_L_ of Mg_3_Sb_2_ alloys. All these features could explain the observed difference of ≈±30% on κ_L_ from one material to another (Figure 4d). Importantly, all Mg_3_Sb_2_–Mg_3_Bi_2_ alloys show a very low κ_L_ of 0.5–1.0 W m^−1^ K^−1^ at working temperatures, largely ensuring a high thermoelectric figure of merit, *zT*. Excitingly, the current work realizes a much higher *zT* than that ever reported at 300–500 K (**Figure** **5**), although the peak *zT* remains comparable to literature results (Figure S6, Supporting Information). Moreover, the high performance is highly reproducible as confirmed in multiple samples (Figure 5; Figures S5d and S6, Supporting Information).

## 3 Conclusion

In summary, this work successfully reveals a high carrier mobility inherent to n‐type Mg_3_Sb_2_ materials, which leads to a discovery of superior thermoelectric performance particularly at 300–500 K. This is enabled by a development in synthesis by tantalum‐sealing and in hot press by deforming/fusing big pieces of ingot materials, for coarse grains with minimal oxidation. This work realizes in Mg_3_Sb_2_ alloys among all known n‐type thermoelectrics the most competitive thermoelectric figure of merit *zT* to that of commercialized Bi_2_Te_3_ in the temperature range of 300–500 K, enabling this material to be a potential sustainable solution for Bi_2_Te_3_ thermoelectrics.

## 4 Experimental Section

*Materials and Methods*: Polycrystalline Mg_3.05_Sb_2−_ *_x_*_−_ *_y_*Bi*_y_*_−_ *_x_*Te*_x_* (*x* ≤ 0.04, *y* ≤ 1.5) samples were synthesized by melting stoichiometric amounts of high purity elemental Mg (99.95%), Bi (99.99%), Te(99.95%), and Sb (99.9%) in sealed tantalum tubes in quartz ampoules at 1373 K for 10 h, quenching in cold water, and annealing at 873 K for 72 h. As a replacement of tantalum, stainless steel was confirmed to be an alternative option, yet a graphite crucible was needed for separating stainless steel from the melt due to the corrosivity of Sb. For a reason of simplicity in reaction container, tantalum‐sealing was used eventually in this work. The sealing of tantalum tubes was done with an arc‐melting system in argon with a pressure slightly lower than one atmosphere. An excess of ≈1.7% Mg (i.e., Mg_3.05_Sb_2_) was used to compensate the loss during the synthesis, because of the high vapor pressure and causticity of Mg. Te‐doping at Sb site was used to tune the electron concentration. A reaction between raw materials with tantalum was not observable, enabling an easy separation between the final products and tantalum tubes. The obtained ingots showed smooth surfaces with metallic gray in color, indicating a full melting in this work. The ingots were cut into relatively big pieces (≈3 mm in diameter or bigger) in an argon‐filled glove box, followed by a transfer as quick as possible to the vacuum chamber of the hot press system for minimal exposure to air, and then an immediate hot‐pressing process. Hot press was carried out in vacuum at 800 K for 25 min under a uniaxial pressure of ≈100 MPa with an induction heating hot press system (Figure 1). As a control experiment, ingots of Mg_3.05_SbBi_0.97_Te_0.03_ were also hand ground into powders either in an argon‐filled glovebox or in air to control the level of oxidation, followed by the same procedure of hot pressing. These enabled materials with different grain sizes and different levels of oxidation for the investigation of size and oxidation effects on thermoelectric properties.

The dense pellet samples, ≈12 mm in diameter and ≈1.5 mm in thickness, were used for measurements. The thermal diffusivity (*D*) was measured using a laser flash technique (Netzsch LFA457) and the thermal conductivity (κ) was calculated via κ = *dC*_p_ *D*, where *d* is the density estimated by the mass/volume method and *C*_p_ is the heat capacity determined by the Dulong–Petit limit and is assumed to be temperature independent. The measurement uncertainty of *S*, ρ, and κ was about 5%. Resistivity (ρ), Hall coefficient (*R*_H_), and Seebeck coefficient (*S*) were measured under a helium atmosphere in the temperature range from 300 to 700 K. The Seebeck coefficient was measured from the slope of the thermopower versus temperature differences within 0–5 K. The resistivity and Hall coefficient were measured using the van der Pauw technique under a reversible magnetic field of 1.5 T. Microstructures were characterized on surfaces polished with ethanol, using a scanning electron microscope (Phenom Pro. and Nova NanoSEM 450) equipped with an energy dispersive spectrometer. A small portion of ingots were hand ground into powders for X‐ray diffraction, and the diffraction data were collected on a DX‐2007 diffractometer using Cu‐Kα radiation (λ = 1.1.5406 Å).

## Conflict of Interest

The authors declare no conflict of interest.

## Supporting information

Supplementary
