# Supplementary material for: Revelation of Inherently High Mobility Enables Mg3Sb2 as a Sustainable Alternative to n‐Bi2Te3 Thermoelectrics
Source: Adv Sci (Weinh). 2019 Jul 13;6(16):1802286. doi: 10.1002/advs.201802286 (PMC6702648; doi:10.1002/advs.201802286)
Supplement: Supplementary file 1 — Supplementary [file ADVS-6-1802286-s001.pdf]

Copyright WILEY-VCH Verlag GmbH & Co. KGaA, 69469 Weinheim, Germany, 2019.

ADVANCED SCIENCE
Open Access

Supporting Information

for *Adv. Sci.,* DOI: 10.1002/advs.201802286

Revelation of Inherently High Mobility Enables Mg_3Sb_2 as a Sustainable Alternative to n-Bi_2Te_3 Thermoelectrics

*Xuemin Shi, Cheng Sun, Zhonglin Bu, Xinyue Zhang, Yixuan Wu, Siqi Lin, Wen Li, Alireza Faghaninia, Anubhav Jain, and Yanzhong Pei**

Supporting Information

Revelation of inherently high mobility enables Mg_3Sb_2 as a sustainable alternative to n-Bi_2Te_3thermoelectrics

Xuemin Shi[1], Cheng Sun[1], Zhonglin Bu[1], Xinyue Zhang[1], Yixuan Wu[1], Siqi Lin[1], Wen Li[1],Alireza Faghaninia[2], Anubhav Jain[2], Yanzhong Pei[1,]*

DFT calculations:

The first-principles calculations were performed using Kohn-Sham density functional theory (DFT)[1] as implemented in the Vienna ab initio simulation package (VASP).[2] We used Heyd-Scuseria-Ernzerh of (HSE)[3] hybrid functional for a more accurate computation of electronic band gaps and projected augmented-wave (PAW).[4] Note that the computation results reported in this work are all calculated with HSE including the structure optimization. Table S1 provides a comparison of experimental vs. PBE vs. HSE relaxed lattice parameters. The band structure calculations were performed using automate workflow management package [5] with the default parameters of version 0.8.2 except for the k-points as 1) they miss the CBM in a conventional line-mode high symmetry k-points path and 2) they are not dense enough for a nice and smooth visualization. For example, the planewave energy cutoff is set to 520 eV and the structure relaxation is performed in a k-point grid of 5×5×3 and exact exchange contribution of AEXX=0.25 in VASP when using the HSE functional. The fermi surface plot is obtained by a combination of the BoltzTraP[6] code and pymatgen[7] plotting tools. In order to get an accurate and smooth fermi surface after the HSE calculations, we used a relatively dense uniform k-point grid consist of 334 k-points. The initial structure of Mg_3SbBi is obtained by replacing one Sb atoms by Bi in the unit-cell of Mg_3Sb_2.[8]

Grain size estimation：

SEM images (left column of **Figure S2**) taken from the polished and etched surfaces of the samples compared in **Figure 1** are processed, and the grains of which are outlined (middle column of **Figure S2**) by the software ImageJ. This software further enables a statistic on grain size distribution and areal cumulation (right column of **Figure S2**). Taking into account the factor that very small grains tend to locate at the interstitials of a few big ones (**Figure S2**) and these small grains actually accumulate to only a small portion of the entire material, big grains that accumulatively contribute to >95% of the material are included for estimating the average circle equivalent diameters. The resultant average circle equivalent diameters of the grains in each material are listed in **Table S2**, which confirms much large grains in hot-deformed sample starting with big pieces of ingot then that of hot-pressed samples starting with hand-ground powders (**Figure 1**).

Supplementary References

[1] P. Hohenberg, W. Kohn, *Phys. Rev.* **1964**, 136, B864.

[2] G. K. J. Furthmuller, *Phys. Rev. B* **1996**, 54, 11169.

[3] J. Paier, M. Marsman, K. Hummer, G. Kresse, I. C. Gerber, J. G. Angyan, *J. Chem. Phys.* **2006**, 124, 154709.

[4] a) G. Kresse, D. Joubert, *Phys. Rev. B* **1999**, 59, 1758; b) P. E. Blöchl, *Phys. Rev. B* **1994**, 50, 17953.

[5] K. Mathew, J. H. Montoya, A. Faghaninia, S. Dwarakanath, M. Aykol, H. Tang, I. Chu, T. Smidt, B.

Bocklund, M. Horton, J. Dagdelen, B. Wood, Z. Liu, J. Neaton, S. Ping, K. Persson, A. Jain, *Comput. Mater. Sci.* **2017**, 139, 140.

[6] G. K. H. Madsen, D. J. Singh, *Comput. Phys. Commun.* **2006**, 175, 67.

[7] S. P. Ong, W. D. Richards, A. Jain, G. Hautier, M. Kocher, S. Cholia, D. Gunter, V. L. Chevrier, K. A. Persson, G. Ceder, *Computational Materials Science* **2013**, 68, 314.

[8] Snyder, G. J.; Toberer, E. S., *Nat.Mater.* **2008,** *7*, 105.

[9] a) K. F. Hsu, S. Loo, F. Guo, W. Chen, J. S. Dyck, C. Uher, T. Hogan, E. K. Polychroniadis, M. G. Kanatzidis, *Science* **2004**, 303, 818; b) L. You, Y. Liu, X. Li, P. Nan, B. Ge, Y. Jiang, P. Luo, S. Pan, Y. Pei, W. Zhang, G. J. Snyder, J. Yang, J. Zhang, J. Luo, *Energy & Environmental Science* **2018**, 11, 1848; c) H. Tamaki, H. K. Sato, T. Kanno, *Adv. Mater.* **2016**, 28, 10182; d) J. Zhang, L. Song, S. H. Pedersen, H. Yin, L. T. Hung, B. B. Iversen, *Nat Commun* **2017**, 8, 13901; e) T. Kanno, H. Tamaki, H. K. Sato, S. D. Kang, S. Ohno, K. Imasato, J. J. Kuo, G. J. Snyder, Y. Miyazaki, *Appl. Phys. Lett.* **2018**, 112; f) X. Shi, J. Yang, J. R. Salvador, M. F. Chi, J. Y. Cho, H. Wang, S. Q. Bai, J. H. Yang, W. Q. Zhang, L. D. Chen, *J. Am. Chem. Soc.* **2011**, 133, 7837; g) X. Chen, H. Wu, J. Cui, Y. Xiao, Y. Zhang, J. He, Y. Chen, J. Cao, W. Cai, S. J. Pennycook, Z. Liu, L.-D. Zhao, J. Sui, *Nano Energy* **2018**, 52, 246; h) H. J. Wu, L.-D. Zhao, F. S. Zheng, D. Wu, Y. L. Pei, X. Tong, M. G.Kanatzidis, J. Q. He, *Nat. Commun.* **2014**, 5, 4515; i) S. Ohno, K. Imasato, S. Anand, H. Tamaki, S. D. Kang, P. Gorai, H. K. Sato, E. S. Toberer, T. Kanno, G. J. Snyder, *Joule* **2018**, 2, 1; j) WeiShu Liu, Qinyong

Zhang, Yucheng Lan, Shuo Chen, Xiao Yan, Qian Zhang, Hui Wang, Dezhi Wang, Gang Chen, Z. Ren, *Adv. Energy Mater.* **2011**, 1, 577; k) Lipeng Hu, Haijun Wu, Tiejun Zhu, Chenguang Fu, Jiaqing He, Pingjun Ying, X. Zhao, *Adv. Energy Mater.* **2015**, 5, 1500411; l) S. Wang, Y. Sun, J. Yang, B. Duan, L. Wu, W. Zhang, J. Yang, *Energy Environ. Sci.* **2016**, 9, 3436; m) R. Shu, Y. Zhou, Q. Wang, Z. Han, Y. Zhu, Y. Liu, Y. Chen, M. Gu, W. Xu, Y. Wang, W. Zhang, L. Huang, W. Liu, *Adv. Funct. Mater.* **2019**, 29, 1807235; n) K. Imasato, S. D. Kang, G. J. Snyder, *Energy & Environmental Science* **2019**, 12, 965; o) J. Shuai, J. Mao, S. Song, Q. Zhu, J. Sun, Y. Wang, R. He, J. Zhou, G. Chen, D. J. Singh, Z. Ren, *Energy & Environmental Science* **2017**, 10, 799; p) J. Mao, Y. Wu, S. Song, Q. Zhu, J. Shuai, Z. Liu, Y. Pei, Z. Ren, *ACS Energy Letters* **2017**, 2245.

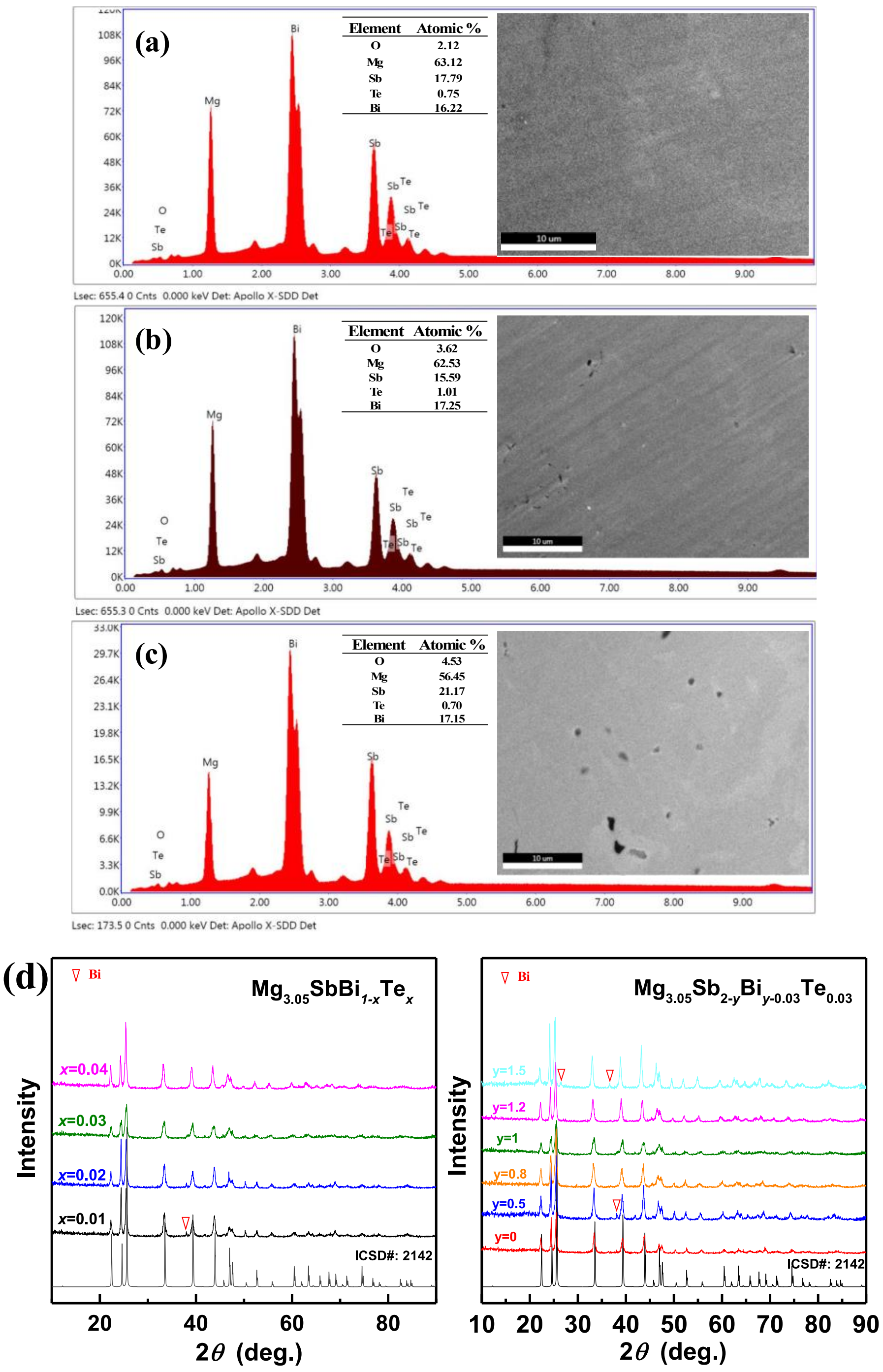

FigureS1.SEM images taken from the polished surface of $Mg_{3.05}SbBi_{0.97}Te_{0.03}$ pellets hot pressed using big pieces (a), hand-ground powders in argon-filled glovebox (b) and in air (c), with compositions determined by EDS. Powder XRD patterns (d) for $Mg_{3.05}Sb_{2-x-y}Bi_{y-x}Te_x$ ($x\leq0.04,y\leq1.5$) at room temperature.

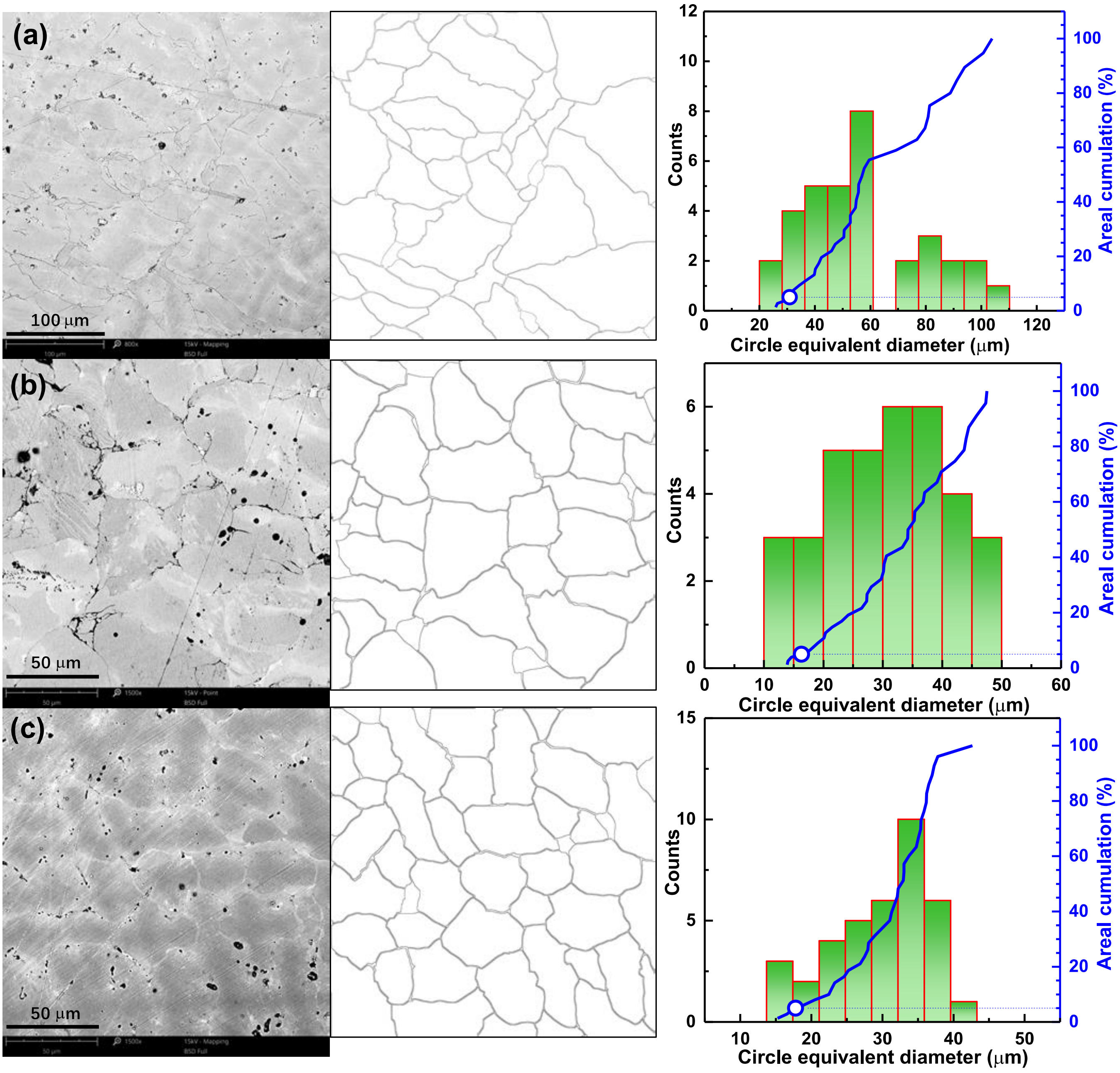

Figure S2. SEM images processed by the software *ImageJ* and the resultant statistics on the grains for $Mg_{3.05}SbBi_{0.97}Te_{0.03}$materials shown in Figure 1.

Table S1. The experimental and computed lattice parameters in angstrom (a=b).

Compound	Experimental [Å]	DFT(GGA-PBE) [Å]	HSE [Å]
Mg_3Sb_2	a=4.559	a=4.598	a=4.582
	c=7.227	c=7.276	c=7.242
Mg_3SbBi	a=4.593	a=4.657	a=4.634
	c=7.299	c=7.381	c=7.325

Table S2. Statistic of grains for the $Mg_{3.05}SbBi_{0.97}Te_{0.03}$samples shown in Figure 1 and Figure S2.

Image #	Grains	Total area [μm^2]	Average area [μm^2]	Average circle equivalent diameter [μm]
(a)	34	99781.8	2934.8	61.1
(b)	35	28722.1	820.6	32.3
(c)	37	27297.3	737.8	30.7

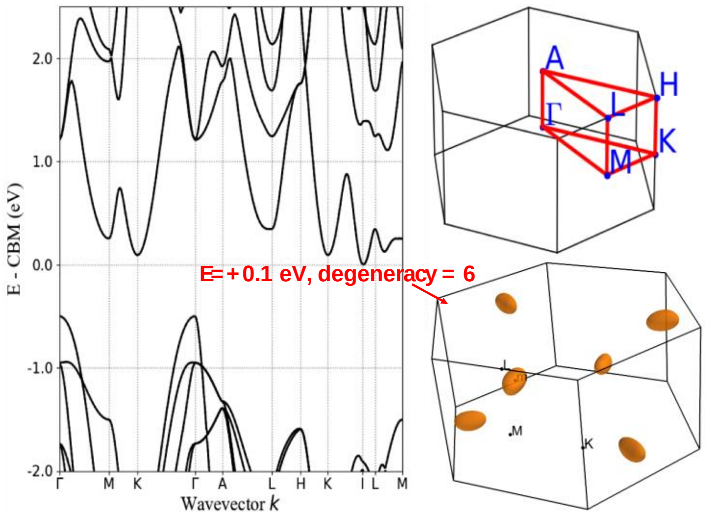

Figure S3. The band structure and fermi surface of Mg_3Sb_2 calculated using HSE functional. The reference energy is set at the conduction band minimum (CBM). Note that the k-point of the CBM is located inside Brillouin Zone (BZ) close to the point L. This point (l) is not one of the high symmetry points on the line path shown on the faces and edges of the BZ. The fermi surface at 0.1 eV above the conduction band minima shows 6 in total roughly spherical electron pockets.

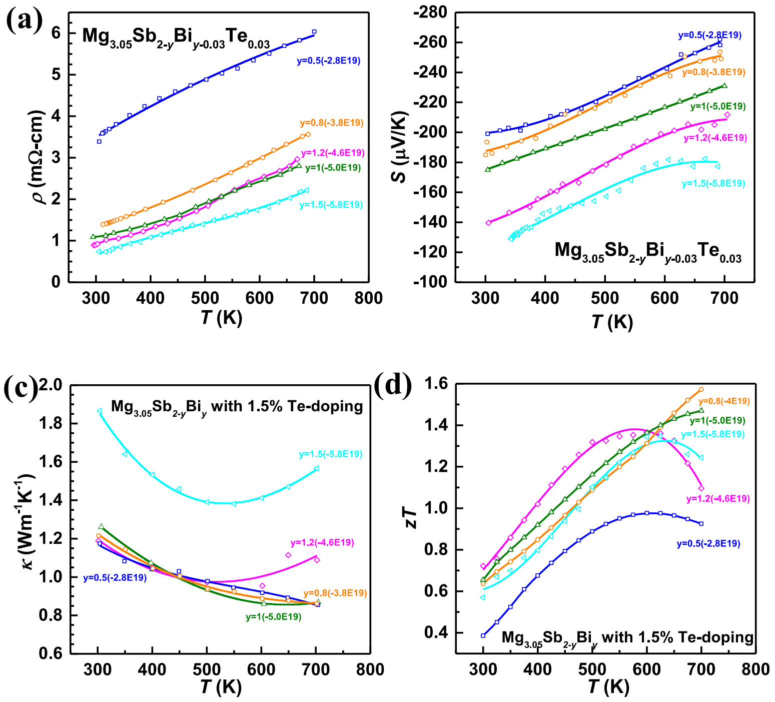

Figure S4. Temperature dependent resistivity (a), Seebeck coefficient (b), thermal conductivity (c) and thermoelectric figure of merit (d) for $Mg_{3.05}Sb_{2-y}Bi_{y-0.03}Te_{0.03}$ ($y \leq 1.5$). The unit of carrier concentration identifying samples is cm^{-3}.

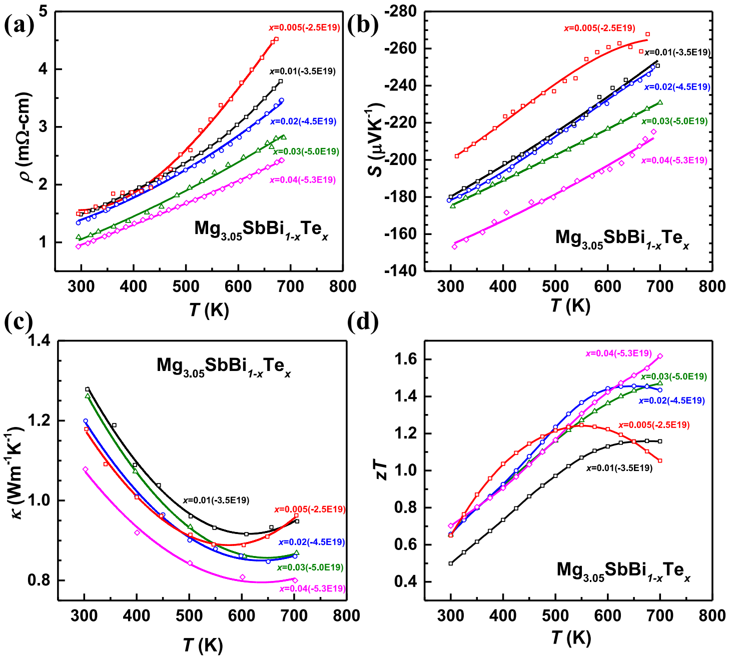

Figure 5. Temperature dependent resistivity (a), Seebeck coefficient (b), thermal conductivity (c) and thermoelectric figure of merit (d) for $Mg_{3.05}SbBi_{1-x}Te_x$ ($x \leq 0.04$). The unit of carrier concentration identifying samples is cm^{-3}.

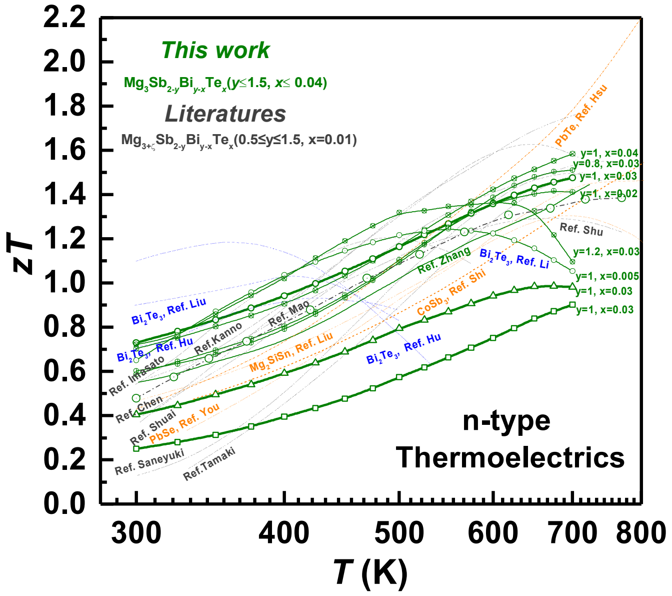

Figure S6. Temperature dependent thermoelectric figure of merit, zT for $Mg_{3.05}Sb_{2-x-y}Bi_{y-x}Te_x$ ($x \leq 0.04, y \leq 1.5$), with a comparison to literature results for n-type thermoelectrics.[9]
